# Antibiotic consumption in India: geographical variations and temporal changes between 2011 and 2019

**DOI:** 10.1093/jacamr/dlac112

**Published:** 2022-10-26

**Authors:** Shaffi Fazaludeen Koya, Senthil Ganesh, Sakthivel Selvaraj, Veronika J Wirtz, Sandro Galea, Peter C Rockers

**Affiliations:** School of Public Health, Boston University, Boston, MA, USA; Public Health Foundation of India, New Delhi, Delhi, India; Public Health Foundation of India, New Delhi, Delhi, India; School of Public Health, Boston University, Boston, MA, USA; School of Public Health, Boston University, Boston, MA, USA; School of Public Health, Boston University, Boston, MA, USA

## Abstract

**Objectives:**

To describe and compare private sector systemic (J01) antibiotic consumption across Indian states from 2011 to 2019.

**Methods:**

We used the nationally representative PharmaTrac dataset to describe the consumption rates in DDD across national, state and state-group [high focus (HF) and non-high focus (nHF)] levels. We used median and IQRs to describe and compare across states and state groups, and relative change and compound annual growth rate (CAGR) to examine temporal changes.

**Results:**

The annual consumption rate decreased by 3.6% between 2011 and 2019. The share of Access antibiotics decreased (13.1%) and the Access/Watch ratio declined from 0.59 to 0.49. State consumption rates varied widely (HF states reported lower rates) and the inappropriate use increased over the years, especially among HF states. The HF and nHF states showed convergence in the share of the Access and the Access/Watch ratio, while they showed divergence in the use of Discouraged fixed-dose combinations.

**Conclusions and implications:**

India’s private-sector antibiotic consumption rate was lower than global rates. The rates varied across states and appropriateness of use decreased in most states over the years. States with an increase in appropriate use over time could serve as best practice examples. Studies to understand the factors affecting inappropriate use are required alongside improved data systems to monitor the public-sector provision of antibiotics to understand the total consumption.

## Introduction

Antibiotic resistance is intrinsically linked to animal and human consumption patterns, partly driven by inappropriate use of antibiotics. In turn, the emergence of antibiotic resistance fuels changes in consumption patterns, as more costly broad-spectrum antibiotics become required to manage even common conditions.^1^

India is the largest consumer of antibiotics globally in terms of absolute volume.^2^ Studies from India have reported poor prescription quality^3^ including unindicated prescription of broad-spectrum antibiotics without evidence of bacterial infection.^4^ These findings are of particular public health relevance considering India reports high antibiotic resistance in bacteria that cause certain common infections.^5^

Wide variations exist between Indian states in terms of their population age structure, health-seeking behaviour, infectious disease burden, health systems organization and the relative contribution of the public and private sector in healthcare.^6^ However, we do not have studies examining differences in antibiotic consumption across Indian states. Understanding these differences helps us to assess the influence of state-specific policies and programmes in antibiotic consumption, inform such policies to improve antibiotic use, set and enforce state-specific targets, correlate with emerging antibiotic resistance patterns, and assess, design and implement effective antibiotic stewardship programmes.^7–9^ Therefore, we aimed to answer the following questions using private-sector antibiotic sales data for the period 2011–2019: (1) What are the national- and subnational-level private-sector antibiotic consumption rates during 2011–19 in India? (2) How much did the private-sector antibiotic consumption vary across Indian states during 2011–19? and (3) What changes occurred in the appropriateness of private-sector antibiotic consumption patterns during 2011–19?

## Materials and methods

We conducted a longitudinal ecological analysis of antibiotic sales in India using the PharmaTrac dataset—a nationally representative drug sales audit dataset—a detailed description of which has been published previously.^10^ Briefly, the data are gathered from a panel of 9000 stockists (60% of total stockists across the country), representing data from 5000 pharmaceutical companies, 18 000 small distributors and substockists, and 500 000 retailers, which includes hospitals, clinics, pharmacies and individual clinicians.^11^ We included only systemic antibacterials (classified under J01 as per the WHO ATC system) in our analysis, and excluded topical preparations, eye/ear drops, gels, pessaries and suppositories, as described previously.^10^

We calculated the annual consumption rate at the national and state level. We analysed consumption across different characteristics, namely WHO AWaRe (Access, Watch, Reserve) groups, product type [fixed-dose combinations (FDCs)/single formulations (SFs)], essentiality [listed/not listed in the national list of essential medicines (NLEM)] and approval by the Central Drugs Standard Control Organization (CDSCO). The details of these groupings, sources of data, and classification have been published previously,^10^ and are summarized in Panel S1 (available as Supplementary data at *JAC* Online). We used oral liquid preparation consumption as a proxy indicator of paediatric oral antibiotic use in the community, and the consumption of injectables as a proxy indicator of in-hospital antibiotic use. We also conducted a subanalysis of the trends in consumption of different generation cephalosporins as a measure to assess the inappropriate use of newer generation antibiotics.

We used DDD, the standard metric to measure consumption volume.^12^ We used the projected mid-year population from the National Population Commission (https://censusindia.gov.in/). The DDD per 1000 persons per day (DID) values were calculated at the national and state level across all the years, and trends were analysed using graphs. We used median, IQR, relative change and compound annual growth rate (CAGR) as summary measures. The details of definitions, methods and formulas are included in the Panel S2.

India’s national health mission classifies states into high-focus (HF) states and non-high-focus (nHF) states based on health infrastructure, life expectancy, fertility rate and child and maternal mortality indicators.^13^ HF states include Bihar, Chhattisgarh, Jharkhand, Madhya Pradesh, Odisha, Rajasthan, Uttar Pradesh and seven northeastern states. Researchers have extensively used this classification of states to study health inequities and the impact of government interventions.^14–16^ We compared population-weighted estimates of DIDs and the relative consumption of AWaRe groups of antibiotics, FDCs, NLEM and approved products across HF and nHF states. We used the Wilcoxon rank-sum test to examine statistically significant differences in the medium values of consumption characteristics between the state groups. Besides, we visually analysed trends using time series graphs. Further, to examine if the consumption pattern varied between states within the same group, we compared key characteristics of antibiotic consumption between states in the HF group of states and between states in the nHF group of states. We used the Kruskal–Wallis rank-sum test to examine statistically significant differences in median values of consumption characteristics between states. *P* values less than 0.05 were considered significant for all analysis. Maps were used to depict relative changes of various indicators at the state level.

We used Excel version 16.0 (Microsoft, 2022), STATA software version 17.0 (Stata Corp LP, 2021) and R software version 4.1.1 (R Core Team, 2020) to clean, organize and analyse the data. All maps were created using STATA version 17.0. We report the study findings as per the strengthening the reporting of observational studies in epidemiology (STROBE) guidelines.

### Ethics

Individual-level data were not collected and there were no personal identifiers in the dataset that we analysed. Therefore, we did not require ethical approval for our study.

## Results

At the national level, total private-sector antibiotic consumption increased by 12.0% between 2011 and 2016, from 4749 to 5358 million DDDs, and then decreased to 5071 million in 2019, registering a net increase of 6.8% between 2011 and 2019 and a CAGR of 0.63%. Annual DIDs at the national level are provided in Table S1(a). The median consumption rate during the period was 10.7 DIDs (IQR 10.6–10.9). The DID increased by 6.5% between 2011 (10.7) and 2016 (11.4) and then decreased by 8.5% between 2016 and 2019 (10.3). Table 1 shows the summary of consumption patterns over the study period. The share of Access group antibiotics decreased during the study period (CAGR = −1.7%), while that of Reserve groups increased (CAGR = 16.8%). The Access/Watch ratio declined by 10 percentage points (0.59 to 0.49). The share of FDCs increased till 2016, before it decreased to the 2011 level in 2019, while the share of NLEM decreased till 2016 and then increased to reach the 2011 level in 2019. The relative share of centrally approved formulations outstripped unapproved formulations in 2018 [Table S1(a)]. While the total oral formulations showed a relative increase of 4.6% between 2011 and 2019, oral liquid preparations alone showed a relative increase of 21.2%, indicating substantial increase in the use of paediatric oral preparations [Table S1(b)]. Although parenteral preparations accounted for a very small proportion (4.9%) of total use in the study period, their use increased by 33.1% in 2019 relative to 2011, indicating substantial increase in the in-hospital antibiotic use [Table S1(c)]. Among cephalosporins, the volume of third-generation molecules consumed was more than the combined volumes of first- and second-generation molecules, which may indicate disproportionately higher use of new-generation antibiotics. (Figure S1).

**Table 1.** Summary of antibiotic consumption at the national level during 2011–19

Measures	Median [IQR]^a^	Relative change (%)	CAGR (%)
DID, absolute value	10.7 [10.6–10.9]	−3.6	−0.5
Access, %	25.9 [25.9–26.2]	−13.1	−1.7
Watch, %	51.8 [49.6–53.1]	3.9	0.5
Reserve, %	0.4 [0.4–0.8]	246.9	16.8
Discouraged, %	19.5 [18.0–20.5]	11.3	1.3
Not classified, %	0.9 [0.9–5.2]	−52.0	−8.8
FDC, %	36.4 [34.5–37.4]	0.9	0.1
NLEM listed, %	43.8 [42.8–45.9]	3.2	0.4
CDSCO approved, %	49.2 [48.4–49.7]	6.2	0.8

The relative change was calculated using values for 2011 and 2019.

a Median and IQR calculated using annual values at the national level.

**Figure 1.**
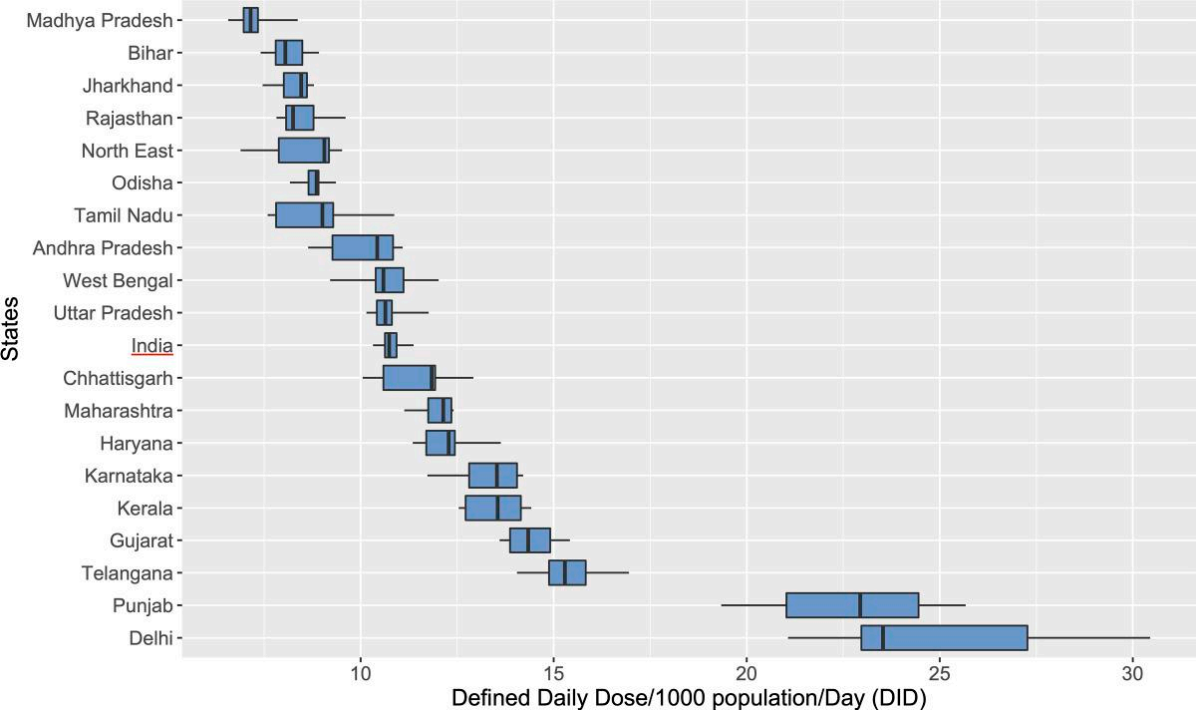
Box-and-whisker plots of private-sector antibiotic use in Indian states, 2011–19.

Figure 1 summarizes consumption during the study period at the state level using box-and-whisker plots. Delhi recorded the highest median consumption rate (DID = 23.5) followed by Punjab (22.9) and Telangana (15.3). The lowest rates were reported in the HF states of Madhya Pradesh (7.2), Bihar (8.1), Rajasthan (8.3), Jharkhand (8.5) and Odisha (8.9). During the 9 years, the rates increased in six states, with Punjab reporting the highest increase (4.3 DIDs), while the rate decreased in 20 states, including the seven northeastern states. The most substantial decline was in Delhi (6.2 DIDs, 22.7%) (Table S2).

**Figure 2.**
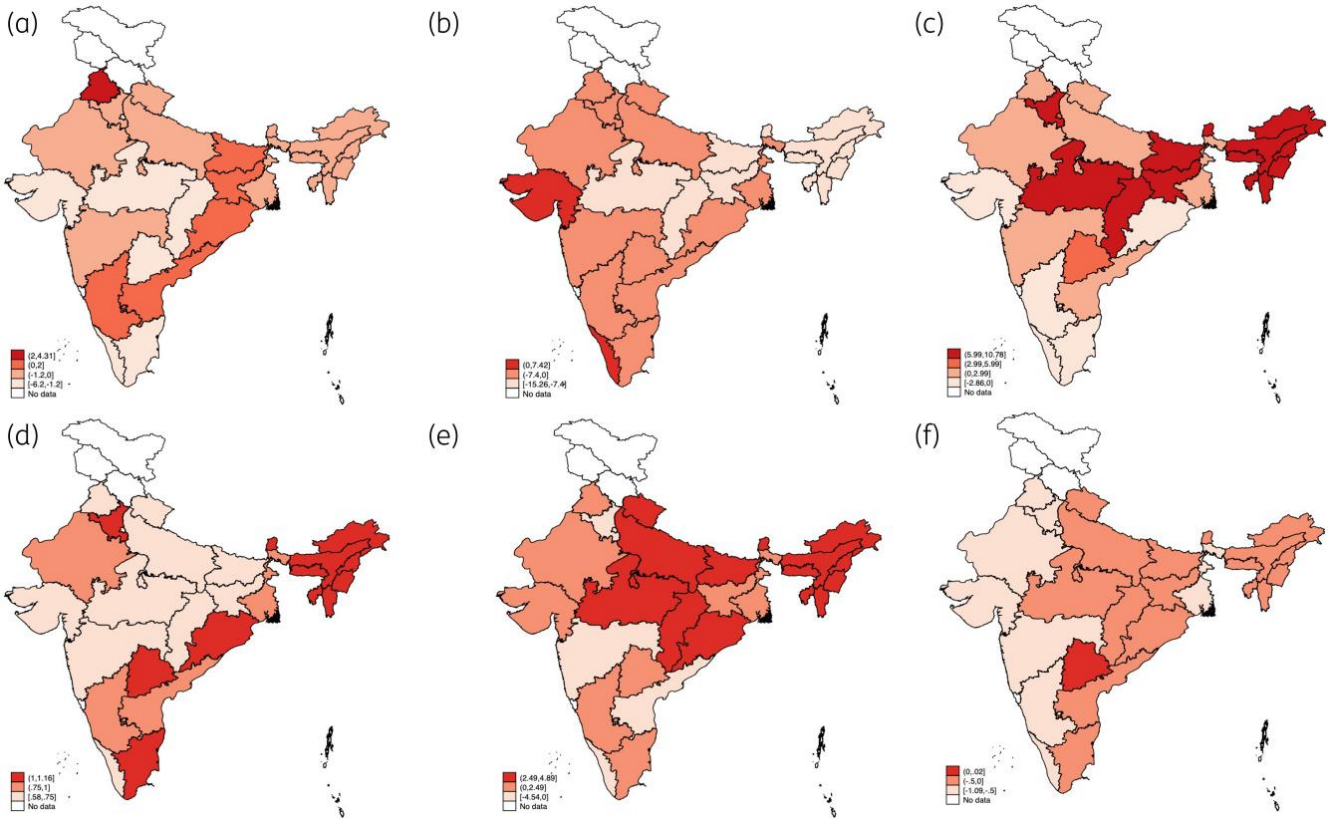
Maps showing changes in private-sector antibiotic use in Indian states, 2011–19. (a) Change in DIDs (absolute values); percentage change in: (b) ‘Access’ antibiotics; (c) ‘Watch’ antibiotics; (d) ‘Reserve antibiotics; (e) ‘Discouraged antibiotics; and (f) ‘Not classified’ antibiotics.

Figure 2 shows changes in DID values and shares of AWaRe groups across states between 2011 and 2019 (Tables S2–S11). The share of the Access group increased in Kerala (7.4%) and Gujarat (1.2%), while it decreased in all other states, and the most substantial decline was in Bihar (13.4%). The Watch group antibiotics increased in all states except Gujarat, Karnataka, Kerala and Tamil Nadu. The use of Reserve antibiotics increased in all states but remained around 1.0% across the study period. The use of Discouraged FDCs decreased in five states, the most substantial absolute decline being 4.5% in Kerala, while the consumption increased in 14 states, more pronounced in HF states. The highest Access/Watch ratio in 2011 was reported from Bihar (0.86), followed by Chhattisgarh and Madhya Pradesh—all HF states. However, the ratio in Bihar declined to 0.46 in 2019, while the ratio in Kerala improved from 0.54 in 2011 to 0.72 in 2019.

HF and nHF states significantly differed in consumption (Table 2). They had significantly different median population-weighted DIDs [HF: 9.1 (IQR  9.0–9.4); nHF:13.0 (IQR  12.9–13.2); *P* < 0.001]; share of Access [HF: 27.1% (IQR  26.7–30.1); nHF: 25.9% (IQR  23.0–26.1); *P* = 0.03], Watch [HF: 48.6% (IQR  46.2–50.6); nHF: 53.8% (IQR  52.4–55.0); *P* < 0.01); and FDCs [HF: 38.6% (IQR  36.7–38.8); nHF: 35.5% (IQR  33.6–36.1; *P* < 0.01). The DID increased from 2011 to 2016 for both groups, from 9.0 to 9.9 for HF states and 13.0 to 13.4 for nHF states. From 2016 to 2019, the rates decreased to 8.8 and 12.4 for HF and nHF states, respectively.

**Table 2.** Comparison of antibiotic consumption (population-weighted) across HF and nHF groups of states, 2011–19

Measure/	Median [IQR]		Relative change^a^		CAGR		*P* value^b^
State groups	HF	nHF	HF	nHF	HF	nHF	
DID	9.1 [9.0–9.4]	13.0 [12.9–13.2]	−2.2%	−5.4%	−0.3%	−0.7%	**<0**.**001**
Access, %	27.1 [26.7–30.1]	25.9 [23.0–26.1]	−24.4%	−2.5%	−3.4%	−0.3%	**0**.**03**
Watch, %	48.6 [46.2–50.6]	53.8 [52.4–55.0]	9.7%	0.7%	1.2%	0.1%	**<0**.**01**
Reserve, %	0.4 [0.32–0.67]	0.5 [0.4–0.79]	400.0%	266.7%	22.3%	17.6%	0.49
Discouraged, %	21.4 [18.6–22.4]	17.9 [17.0–19.4]	23.2%	1.4%	2.6%	0.2%	0.3
Not classified, %	0.6 [0.55–4.2]	1.2 [1.1–6.0]	−50.0%	−42.9%	−8.3%	−6.8%	0.06
FDC, %	38.6 [36.7–38.8]	35.5 [33.6–36.1]	−5.5%	6.3%	−0.7%	0.8%	**<0**.**01**
NLEM listed, %	43.2 [42.2–44.2]	44.8 [43.8–47.2]	2.6%	3.5%	0.3%	0.4%	0.08
CDSCO Approved, %	50.1 [48.8–51.1]	47.9 [47.1–49.5]	4.1%	9.0%	0.5%	1.1%	0.16

a The relative change was calculated using values for 2011 and 2019.

b Significance tested for the difference in measures between HF and nHF states using Wilcoxon rank-sum test with continuity correction. Null hypothesis: no difference in median consumption level between the state groups. Alternative hypothesis: the median consumption level differs between the state groups. *P*-values in bold indicate statistically significant differences.

Table 3(a–b) shows the differences in key consumption characteristics across states. There was statistically significant difference between the eight HF states [Table 3(a)] in Access/Watch ratio (*P* = 0.007), percentage share of Access (*P* = 0.002), Reserve (*P* = 0.002), Discouraged (*P* < 0.001), FDC (*P* = 0.008) and non-NLEM (*P* = 0.003) formulations. *Post hoc* analysis showed that Madhya Pradesh and Rajasthan had substantially low Access/Watch ratios and Uttar Pradesh/Uttarakhand had substantially high Access consumption. The 11 HF states significantly differed in Access/Watch ratio, percentage share of Access, Discouraged, FDC and non-NLEM formulations; all *P* < 0.001. These states did not differ in consumption of Reserve antibiotics (*P* = 0.3).

**Table 3.** Comparison of key characteristics of antibiotic consumption across Indian states

State	A/W ratio	Access, %	Reserve, %	Discouraged, %	FDC, %	Non-NLEM, %
HF states						
* *Overall (*n* = 8) states, 9 years)	0.6 (0.5–0.6)	26.3 (24.5–30.0)	0.5 (0.3–0.7)	21.5 (18.6–24.0)	39.7 (36.8–41.9)	56.1 (53.6–58.5)
* *Bihar	0.6 (0.6–0.8)	27.2 (25.9–32.6)	0.2 (0.2–0.4)	23.9 (21.1–25.9)	40.4 (37.8–41.1)	59.2 (55.5–60.0)
* *Chhattisgarh	0.6 (0.5–0.6)	27.8 (25.0–28.7)	0.3 (0.3–0.5)	21.3 (20.2–21.8)	41.8 (38.5–44.0)	55.2 (54.3–56.5)
* *Jharkhand	0.6 (0.5–0.7)	25.2 (23.8–30.5)	0.3 (0.2–0.5)	26.0 (22.8–27.4)	40.1 (38.8–42.4)	57.4 (55.9–58.2)
* *Madhya Pradesh	0.5 (0.4–0.5)	25.2 (24.1–26.4)	0.4 (0.3–0.6)	21.5 (18.6–21.6)	41.2 (38.7–42.2)	56.0 (54.5–57.0)
* *Northeast	0.5 (0.5–0.7)	27.5 (25.7–34.3)	0.6 (0.5–0.9)	18.3 (15.5–19.7)	41.5 (39.6–42.8)	51.5 (50.1–51.7)
* *Odisha	0.6 (0.5–0.6)	25.4 (24.8–26.1)	0.7 (0.6–1.0)	26.3 (23.0–26.4)	39.5 (36.3–39.8)	55.9 (54.2–59.8)
* *Rajasthan	0.5 (0.4–0.5)	24.2 (23.6–24.7)	0.7 (0.5–0.9)	21.7 (17.9–23.0)	40.0 (37.3–41.6)	57.1 (55.8–59.1)
* *Uttar Pradesh	0.6 (0.6–0.7)	30.9 (28.6–33.1)	0.4 (0.3–0.6)	19.0 (17.7–20.1)	34.4 (34.1–35.2)	57.2 (56.6–58.6)
* P* value^a^	0.007	0.002	0.002	<0.001	0.008	0.003
nHF states						
* *Overall (*n* = 11) states, 9 years)	0.5 (0.4–0.5)	25.3 (22.5–27.9)	0.5 (0.4–0.8)	17.9 (15.4–20.2)	35.0 (32.7–37.2)	53.7 (51.5–56.4)
* *Andhra Pradesh	0.5 (0.4–0.5)	25.8 (22.6–27.7)	0.5 (0.4–0.9)	19.4 (17.9–22.1)	34.6 (34.3–35.1)	55.1 (54.2–56.4)
* *Delhi	0.5 (0.4–0.5)	25.5 (24.2–27.5)	0.8 (0.6–1.0)	17.4 (16.5–18.7)	38.1 (36.6–40.9)	52.9 (51.3–55.1)
* *Gujarat	0.4 (0.3–0.4)	21.1 (17.0,21.2)	0.5 (0.5,0.8)	20.2 (18.6,21.2)	35.4 (33.2,36.2)	56.5 (54.2,60.5)
* *Haryana	0.6 (0.5–0.7)	30.9 (27.1–31.5)	0.6 (0.5–0.9)	16.2 (15.3–17.0)	35.0 (34.0–37.0)	52.3 (50.4–52.9)
* *Karnataka	0.5 (0.4–0.5)	24.6 (20.8–25.4)	0.4 (0.3–0.8)	20.2 (18.6–20.5)	37.8 (35.8–40.3)	56.3 (54.9–56.4)
* *Kerala	0.6 (0.5–0.7)	33.2 (26.6–36.1)	0.4 (0.3–0.6)	13.6 (10.3–15.2)	31.0 (30.7–31.8)	45.5 (43.6–48.7)
* *Maharashtra	0.4 (0.3–0.4)	24.4 (17.9–25.0)	0.5 (0.3–0.7)	18.1 (16.8–19.8)	35.0 (33.2–36.6)	57.7 (54.8–58.9)
* *Punjab	0.4 (0.4–0.4)	23.9 (23.3–25.2)	0.4 (0.4–0.6)	17.1 (16.4–18.7)	33.2 (32.7–37.1)	52.6 (52.2–53.3)
* *Tamil Nadu	0.6 (0.5–0.6)	30.7 (26.0–31.8)	0.6 (0.3–0.9)	14.1 (12.6–15.4)	32.4 (31.5–34.5)	52.2 (51.1–53.6)
* *Telangana	0.4 (0.4 –0.4)	21.4 (21.2–21.8)	0.5 (0.4–1.0)	21.6 (21.1–23.2)	38.1 (35.9–39.8)	57.5 (56.8–59.4)
* *West Bengal	0.6 (0.5–0.6)	27.6 (26.5–29.8)	0.5 (0.4–0.6)	18.3 (18.0–20.2)	35.6 (34.5–36.1)	54.5 (51.6–55.5)
* P* value^a^	<0.001	<0.001	0.3	<0.001	<0.001	<0.001

A/W ratio: Access/Watch ratio. All values given as median (IQR).

a Significance tested for the difference in measures between HF and nHF states using Kruskal–Wallis rank-sum test. Null hypothesis: all medians are equal. Alternative hypothesis: at least one median is different.

**Figure 3.**
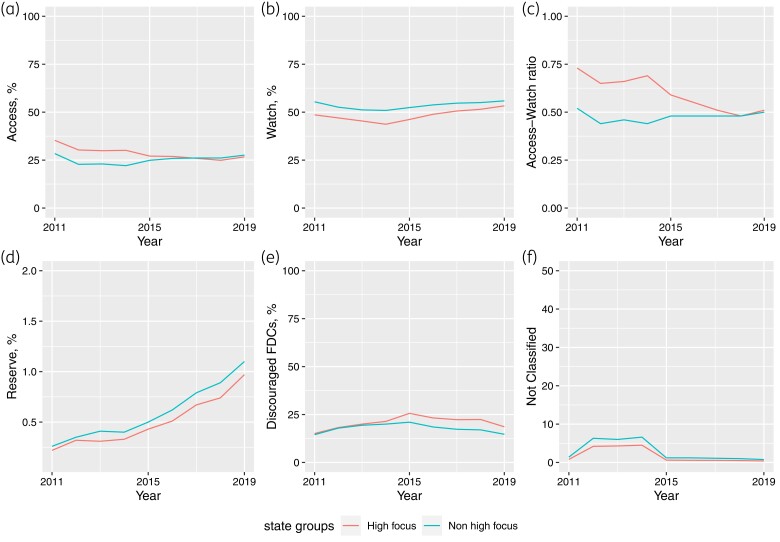
Time series plots showing changes in relative share of: (a) ‘Access’ antibiotics; (b) ‘Watch’ antibiotics; (c) Access/Watch ratio; (d) ‘Reserve antibiotics; (e) ‘Discouraged antibiotics; and (f) ‘Not classified’ antibiotics, in HF and nHF states, 2011–19. The *y*-axes do not have the same scale.

Figure 3(a–f) shows the time series plots of changes in the relative share of AWaRe groups and Access/Watch ratio in HF and nHF states. The share of Access group decreased by 24.4% in HF states between 2011 and 2019, compared with only 2.5% in nHF states. Tables S12–S20 show the consumption patterns at the state-group level for each year. The CAGR of Reserve group antibiotics share was similar (around 17.6%) in HF and nHF states, with the proportional share reaching around 1.0% of total DIDs by 2019 compared with 0.25% in 2011.

Figure S2 shows the differences and trends in FDCs, NLEM formulations and CDSCO-approved products across HF and nHF states. The share of FDCs showed an increasing trend till 2014–15, followed by a decreasing trend in subsequent years. The CAGR over the study period was negative (−0.7%) for HF states and positive (0.8%) for nHF states. On average, close to two-thirds of antibiotics consumed in HF and nHF states were single formulations. The share of NLEM formulations remained below 50% in HF and nHF for most of the years. Close to 50.0% of formulations had no CDSCO approval in HF and nHF states for most of the study period.

## Discussion

This is the first subnational level temporal analysis of antibiotic consumption in India using DDD metrics and the AWaRe system. We used nationally representative private drugs sales data for 2011–19 and reported several findings that have policy implications. Our analysis shows that India’s private-sector antibiotic consumption rate has decreased since 2016, that the rates and consumption patterns vary across states, and that there is a significant level of inappropriate use of broad-spectrum antibiotics with implications for antimicrobial resistance.

First, the antibiotic consumption rate started to decrease after 2016 nationwide and in twelve states, but the most significant changes happened in Delhi and Tamil Nadu (30% reduction). In these states, the trend may be attributable to the improved public-sector provision of drugs—through already existing state medical services corporation (Tamil Nadu)^17^ and newly established Mohalla (community) clinics (Delhi).^18^ The role of 2014 regulatory reforms in the overall decrease in antibiotic use needs to be further studied although some evidence already exists.^19^ Besides, starting in 2016–17 there was a marked increase in the number of government-sponsored generic medicines pharmacy stores (Janaushadhi stores)^20^ that procure drugs through an open government tender. The nationwide demonetization of Indian currency that happened in November 2016 may also have had a role, possibly through a reduction in the spending capacity of the population due to a negative transitory shock resulting from the decline in income and employment.^21^

Second, wide variation exists between states at different levels of socioeconomic and health achievements. This is similar to the spatial disparities reported by a recent study.^22^ The consumption rate varied between 7.0 DIDs for Madhya Pradesh in 2013 and 2015 and 30.5 for Delhi in 2013. The nHF states with high per-capita income like Delhi, Punjab, Telangana, Gujarat and Kerala reported the highest median consumption rates. The only exception to this group was Tamil Nadu, which showed low private consumption despite being an nHF state. State-specific policies and programme interventions and the magnitude of the effect of infectious disease burden, vaccination and social, economic, behavioural and health-financing factors may have played a role in the varying consumption rates. A recent global study had shown a significant positive association between per-capita gross domestic product and antibiotic use.^7^ Studies exploring the factors affecting antibiotic use, cross-learning between states on context-specific best practices, and better surveillance systems to gather data from the private and public sectors are steps forward to improve antibiotic use.

Third, HF states with poor medical and health infrastructure, poor public spending on health, and poor health indicators, including high infectious diseases burden, reported the lowest private consumption rates. Further, even within the same group of states, there were significant differences in consumption patterns. The differences between the states are as striking as the differences reported between countries in previous studies.^23^ The European Surveillance of Antimicrobial Consumption Network (ESAC-Net), with 29 countries, reported an intercountry DID range of 9.7–34.1, and the WHO Europe Antimicrobial Medicines Consumption (AMC) Network, with 15 countries, reported an intercountry DID range of 8.9–30.9.^23^ Notwithstanding that these numbers from Europe also include public-sector data, the comparison is still as relevant as it was previously,^24^ considering that a significant share of antibiotic consumption in India is from the private sector although the public-sector share might have increased in recent years in many states. The median consumption rate for Delhi (23.5) was three times higher compared with that of HF states like Bihar, Rajasthan, Jharkhand and Madhya Pradesh. To put this in perspective, the rate for Delhi is similar to that of Belgium and Italy, and that of Gujarat is similar to that of Sweden and Germany,^23^ while the rate in Madhya Pradesh is lower than that in Côte d’Ivoire and Peru.^8^ The reasons for these differences and the decrease in private consumption after 2016 need to be studied further.

Fourth, there is a significant level of inappropriate consumption of antibiotics including increased use of new-generation antibiotics. Moreover, the inappropriate use, reflected by the higher proportion of Watch and Discouraged FDC antibiotics is higher among the HF states. Additionally, this trend has been increasing in most states, especially the HF states. The HF and nHF states showed convergence in the share of the Access group (in 2017) and Access/Watch ratio (in 2018), while they showed divergence in the use of Discouraged FDCs from 2013. None of the states have achieved the WHO-recommended 60% Access group consumption rate while studies showed that 14 of 29 countries in ESAC-Net countries have achieved the target.^23^ Moreover, heavily populated HF states with a young population, like Bihar and Madhya Pradesh, that started with an Access share of around 40% in 2011 showed a significant decrease (∼35%–40%) over the 9 years. The rapid decline in Access/Watch ratios in HF states is a serious concern as these states have a considerable population share and they lag in socioeconomic and health indicators.

Fifth, the antibiotic consumption we observed in our analysis corresponds to India’s antibiotic resistance patterns. India reports the highest antimicrobial resistance in all years and for all pathogens for which data were available.^25^ The aggregated resistance rates ranged from 17% for aminoglycosides in 2017 to 100% for polymyxins in 2019. In 2018, 87% of isolates detected were resistant to aminopenicillins, while 83% isolates detected in 2019 were resistant to third-generation cephalosporins. Further, India has the worst drug resistance index (DRI) in 2019—a composite index that combines measurements of antibiotic consumption and resistance across multiple pathogen combinations to create a single metric that represents an aggregate level of drug resistance.^26^ These data show that India has the lowest level of antibiotic effectiveness among all the countries analysed. The consumption patterns observed in our analysis concur with observations from these studies—increasing consumption of broad-spectrum Watch antibiotics, including cephalosporins, correlates with a comparatively higher rate of resistance to these antibiotics. Among four states that showed some decrease in Watch share, Kerala was the only state with an improvement in Access/Watch ratio. This may be partially attributed to the antimicrobial stewardship programme involving the private sector that Kerala has undertaken for many years.^27^ Kerala’s policies could serve as an example for other states in India to tackle inappropriate antibiotic use and antimicrobial resistance.

Lastly, we see some significant changes in the use of Discouraged FDCs, NLEM-listed and CDSCO-approved formulations. The sustained reduction in the share of Discouraged FDCs may be attributed to the restrictions in the sale of some FDCs in 2014 and to the ban of over-the-counter sales of some FDCs in 2018. After an 11% reduction between 2011 and 2015, the share of NLEM antibiotics increased by 16% from 2015 to 2019. This may be attributed to the revision of the NLEM in 2015, with different formulations of five new antibiotics added to the list.^28^ The share of CDSCO-approved formulations increased by 11% between 2016 and 2019 after decreasing by 5% between 2011 and 2016. This needs further examination as to whether the unapproved formulations were taken off the market or more inappropriate formulations were approved.

### Limitations

There are some key limitations to our analysis. First, the PharmaTrac data do not capture public-sector consumption. However, 85%–90% of all drug consumption in India happens in the private sector.^29^ Second, although PharmaTrac is nationally representative and extrapolated to reflect the overall medicine sales in the Indian private-sector retail segment, the data do not provide separate consumption data for a few territories. Third, the PharmaTrac data do not differentiate between community and hospital use as they are aggregated at stockist level. But as per the sampling scheme, 85% of data collected by PharmaTrac represent community consumption.^30^ However, even this does not differentiate over-the-counter sales of antibiotics—which is common in India—from prescription-based sales. To estimate the over-the-counter sales, we would need primary survey data at the point of sales, household or population level, which were not available in our dataset. Fourth, the dataset does not provide consumption data segregated by cities, towns and rural areas, which makes it difficult to assess differences in consumption across these geographical areas. Fifth, the appropriateness of prescription at the patient level and whether the consumption reflects the actual demand cannot be ascertained without additional data including prescription data. However, the use of AWaRe and FDC metrics provides valuable insights regarding inappropriateness. Also, the significantly lower consumption rate in HF states with high infectious diseases burden may indicate antibiotic access issues. Finally, an inherent limitation of the WHO DDD method^8^ makes it difficult to differentiate consumption among adults and children as adult antibiotic dosage are used to prepare DDD unit values.

### Conclusions

Using nationally representative medicines sales data, we report significant temporal and geographical variations in the antibiotic consumption rates in India. The consumption rate increased in the early years of the study period, followed by a sustained decline from 2016. However, this decline may not indicate that the needs are met across the states and may even indicate more inappropriate use given the decline in the use of Access molecules and increase in the use of Watch and Reserve molecules—more so in the HF states. The significant variations of inappropriate use at the state level called for state-specific studies to understand the factors—at the health-system, provider and patient level—that drive inappropriate use. States in which appropriate use of antibiotics increased over time could serve as best practice examples. Our findings reiterate the need to strengthen regulations around dispensing of antibiotics and expand the NLEM to make appropriate formulations affordable, besides improving the public-sector drugs provision and antibiotic stewardship programme.

## Supplementary Material

dlac112_Supplementary_Data

## Data Availability

The PharmaTrac data used in this study are available from the AIOCD Pharma soft tech AWACS Pvt. Ltd. Restrictions apply to the availability of these data, which were used under license for this study. Permission can be obtained at https://aiocdawacs.com.

## Transparency declarations

V.J.W. served as co-leader (unpaid) of Health Systems Global Technical Working Group ‘Medicines in Health Systems’ during 2019–21 and leader (unpaid) of the Special Interest Group ‘Drug Utilization and Health Service Research’ during 2018–19. V.J.W. reported research grants (outside the submitted work) from Gilead Sciences, Management Sciences for Health, Sandoz, Fleming Fund, Roche, Amgen, United States Pharmacopeia (USP) and International Volkswagen/Novo Nordisk Fondon/Caixa: Mobility. P.C.R. reported research grants (outside the submitted work) from Gilead Sciences, F. Hoffmann-La Roche, Amgen, Novartis and International Federation for Pharmaceutical Manufacturers and Associations during the past 36 months. S.F.K.’s salary from Boston University is partly supported by the 3-D Commission project under a Rockefeller Foundation grant (2019 HTH 024). All other authors: none to declare.

## Supplementary data

Panels S1 and S2, Figures S1 and S2 and Tables S1–S20 are available as Supplementary data at *JAC* Online.
