# Supplementary material for: Antibiotic consumption in India: geographical variations and temporal changes between 2011 and 2019
Source: JAC Antimicrob Resist. 2022 Oct 26;4(5):dlac112. doi: 10.1093/jacamr/dlac112 (PMC9596537; doi:10.1093/jacamr/dlac112)
Supplement: dlac112_Supplementary_Data [file dlac112_supplementary_data.docx]

**Antibiotic consumption in India: geographic variations and temporal changes between 2011 and 2019**

**Shaffi Fazaludeen Koya^*^, Senthil Ganesh, Sakthivel Selvaraj, Veronika J. Wirtz, Sandro Galea, Peter C. Rockers**

**Supplementary materials**

[*fmshaffi@bu.edu](mailto:*fmshaffi@bu.edu)

**Panel S1: Classification and grouping of antibiotics.**

| ***WHO AWaRe****:* WHO advocates the use of AWaRe classification to monitor the appropriate use of antibiotics. The Access group broadly includes the narrow-spectrum antibiotics (e.g., amoxycillin) recommended as first-line or second-line antibiotics. The Watch consists of broad-spectrum antibiotics (e.g., azithromycin) with a high chance of resistance, to be used only for specific indications. The Reserve group (e.g., colistin) lists last-resort antibiotics. Besides 102 FDCs antibiotics (e.g., ciprofloxacin/ornidazole) are classified Discouraged group. We used the updated WHO 2021 list available at <https://aware.essentialmeds.org/groups>.  ***FDCs:*** Formulations with two or more drugs in a fixed ratio, of which at least one is an antibiotic, as against single formulation (SF).  ***NLEM:*** We used the 2011 list of NLEM for the period 2011-2014 and the 2015 list for the later years to classify antibiotics into NLEM listed (listed) or NLEM not-listed (not listed).  ***CDSCO approval****:* CDSCO is India's national regulatory body for pharmaceuticals and medical devices, and approval details are available every year from 2001 onwards till 2019 from [https://cdsco.gov.in/opencms](https://cdsco.gov.in/opencms/opencms/en/Approval_new/Approved-New-Drugs/). We used this list to classify products as “approved” or “unapproved”. |
| --- |

**Panel S2: Detailed description of calculation**

We calculated DDDs using the following formula.

$$Total DDDs Consumed=\frac{Strength*Pack Size*Packs Consumed}{DDD of Molecule/formulation}$$

From this, we derived the DDD per 1000 persons per day, a standard unit used in global literature (DID) to measure the per-population consumption rate, as follows:

$$DIDs=\frac{Total DDDs Consumed}{Population in thousands*365}$$

We used the projected mid-year population for all the years based on the 2011 census obtained from the National Population Commission (<https://censusindia.gov.in/>) for calculations. The DID values were calculated at the national and state level across all the years, and trends were analyzed. We used median, interquartile range, relative change, and compound annual growth rate (CAGR) as summary measures. Relative change between 2011 and 2019 expressed in percentage, was calculated as below.

$$Relative change=\frac{{Value}_{2019} - {Value}_{2011}}{{Value}_{2011}}$$

CAGR is the average annual change across the years, calculated as

$$CAGR=\left\{ \left( \frac{{Value}_{2019}}{{Value}_{2011}} \right)^{1/8}- 1 \right\}$$

Table S1-A: AWaRe, FDC, NLEM, and CDSCO approval characteristics of antibiotic use, India, 2011-2019

| Year | DID | Access, % | Watch, % | Access to watch ratio | Reserve, % | Discouraged FDCs, % | Not Classified, % | FDC, % | NLEM, % | CDSCO approved,% |
| --- | --- | --- | --- | --- | --- | --- | --- | --- | --- | --- |
| 2011 | 10.7 | 31.0 | 52.7 | 0.59 | 0.28 | 14.8 | 1.2 | 33.6 | 47.4 | 49.3 |
| 2012 | 10.9 | 25.9 | 50.3 | 0.51 | 0.37 | 18.0 | 5.4 | 34.5 | 43.7 | 49.2 |
| 2013 | 10.7 | 25.9 | 48.9 | 0.53 | 0.37 | 19.6 | 5.2 | 36.4 | 42.8 | 48.4 |
| 2014 | 10.9 | 25.7 | 47.8 | 0.54 | 0.37 | 20.5 | 5.6 | 38.5 | 42.6 | 47.8 |
| 2015 | 11.3 | 26.0 | 49.6 | 0.52 | 0.44 | 23.0 | 1.0 | 39.4 | 42.1 | 46.8 |
| 2016 | 11.4 | 26.2 | 51.8 | 0.51 | 0.62 | 20.6 | 0.88 | 37.4 | 43.8 | 49.1 |
| 2017 | 10.6 | 25.9 | 53.1 | 0.49 | 0.75 | 19.5 | 0.85 | 36.9 | 45.4 | 49.7 |
| 2018 | 10.5 | 25.5 | 53.6 | 0.48 | 0.86 | 19.4 | 0.76 | 36.1 | 45.9 | 50.8 |
| 2019 | 10.4 | 27.1 | 54.9 | 0.49 | 0.97 | 16.5 | 0.58 | 33.9 | 48.9 | 52.4 |
| Median | 10.7 | 25.9 | 51.8 | 0.5 | 0.4 | 19.5 | 1.0 | 36.4 | 43.8 | 49.2 |

Table S1-B: Share of liquid vs oral preparations of antibiotics and relative changes, India, 2011-2019

| Form(DDD in million)/year | 2011 | 2012 | 2013 | 2014 | 2015 | 2016 | 2017 | 2018 | 2019 | relative change |
| --- | --- | --- | --- | --- | --- | --- | --- | --- | --- | --- |
| Liquids | 326.64 | 321.66 | 320.76 | 350.39 | 324.4 | 364.14 | 357.43 | 374.13 | 395.96 | 21.2 |
| Solids | 4211.19 | 4336.68 | 4299.78 | 4426.09 | 4659.87 | 4730.85 | 4459.6 | 4420.9 | 4349.91 | 3.3 |
| Total oral forms | 4537.83 | 4658.34 | 4620.54 | 4776.48 | 4984.27 | 5094.99 | 4817.03 | 4795.03 | 4745.87 | 4.6 |

Table S1-C: Share of oral vs parenteral preparations of antibiotics and relative changes, India, 2011-2019

| Form(DDD in million)/year | 2011 | 2012 | 2013 | 2014 | 2015 | 2016 | 2017 | 2018 | 2019 | relative change |
| --- | --- | --- | --- | --- | --- | --- | --- | --- | --- | --- |
| Oral | 4537.83 | 4658.34 | 4620.54 | 4776.48 | 4984.27 | 5094.99 | 4817.03 | 4795.03 | 4745.87 | 4.6 |
| Parenteral | 210.74 | 234.32 | 227.24 | 237.99 | 250.4 | 262.7 | 246.58 | 260.36 | 280.57 | 33.1 |
| Grand Total | 4748.57 | 4892.66 | 4847.78 | 5014.47 | 5234.67 | 5357.69 | 5063.61 | 5055.39 | 5026.44 | 5.9 |

Figure S1: Trends in consumption of first-,second-,third-, and fourth- generation cephalosporins in India, 2011-2019

Table S2: Median DID during 2011-2019, and DID across years, state level

| states/years | Median DID | 2011 | 2012 | 2013 | 2014 | 2015 | 2016 | 2017 | 2018 | 2019 |
| --- | --- | --- | --- | --- | --- | --- | --- | --- | --- | --- |
| Bihar | 8.1 | 7.4 | 7.8 | 7.7 | 8.5 | 8.7 | 8.9 | 7.9 | 8.1 | 8.5 |
| Chhattisgarh | 11.8 | 11.9 | 11.9 | 11.8 | 12.2 | 11.4 | 12.9 | 10.6 | 10.1 | 10.5 |
| Jharkhand | 8.5 | 7.5 | 8.0 | 7.7 | 8.5 | 8.6 | 8.4 | 8.8 | 8.5 | 8.8 |
| Madhya Pradesh | 7.2 | 8.4 | 7.4 | 7.0 | 7.3 | 7.0 | 7.2 | 6.6 | 7.1 | 7.2 |
| Northeast | 9.1 | 7.9 | 9.2 | 9.2 | 9.4 | 9.5 | 9.1 | 8.3 | 7.8 | 6.9 |
| Odisha | 8.9 | 8.2 | 8.7 | 8.2 | 8.9 | 8.9 | 9.4 | 8.9 | 9.0 | 8.8 |
| Rajasthan | 8.3 | 8.2 | 8.3 | 8.1 | 8.8 | 9.6 | 9.3 | 8.4 | 8.0 | 7.8 |
| Uttar Pradesh | 10.6 | 10.4 | 10.8 | 10.8 | 10.6 | 11.6 | 11.8 | 10.2 | 10.5 | 10.2 |
| Andhra Pradesh | 10.4 | 9.3 | 9.5 | 8.6 | 8.7 | 10.8 | 10.4 | 10.9 | 11.1 | 10.8 |
| Delhi | 23.5 | 27.3 | 27.7 | 30.5 | 27.2 | 23.5 | 23.1 | 23.0 | 22.7 | 21.1 |
| Gujarat | 14.3 | 15.3 | 14.6 | 14.3 | 14.9 | 13.9 | 15.4 | 13.8 | 14.1 | 13.6 |
| Haryana | 12.3 | 12.4 | 11.4 | 12.4 | 11.9 | 13.1 | 13.6 | 11.7 | 12.3 | 11.7 |
| Karnataka | 13.5 | 11.7 | 12.8 | 13.5 | 13.8 | 14.2 | 14.1 | 14.1 | 12.8 | 13.5 |
| Kerala | 13.6 | 14.4 | 13.5 | 12.7 | 12.5 | 14.3 | 13.6 | 14.2 | 13.9 | 12.7 |
| Maharashtra | 12.1 | 12.4 | 12.4 | 11.8 | 12.4 | 11.1 | 12.2 | 12.1 | 12.1 | 11.7 |
| Punjab | 22.9 | 19.3 | 20.5 | 21.0 | 22.0 | 25.0 | 25.7 | 24.5 | 22.9 | 23.7 |
| Tamil Nadu | 9.0 | 10.9 | 10.3 | 9.3 | 9.0 | 9.2 | 7.8 | 8.2 | 7.8 | 7.6 |
| Telangana | 15.3 | 16.4 | 17.0 | 15.6 | 15.8 | 15.3 | 14.9 | 14.3 | 14.1 | 15.0 |
| West Bengal | 10.6 | 10.4 | 11.1 | 10.6 | 10.6 | 12.0 | 12.0 | 10.6 | 9.8 | 9.2 |

Table S3: Share of Access formulations across years, state level

| states/years | 2011 | 2012 | 2013 | 2014 | 2015 | 2016 | 2017 | 2018 | 2019 |
| --- | --- | --- | --- | --- | --- | --- | --- | --- | --- |
| Bihar | 38.1 | 32.9 | 32.4 | 32.7 | 27.1 | 27.3 | 25.9 | 24.0 | 24.6 |
| Chhattisgarh | 37.5 | 25.0 | 28.7 | 28.0 | 29.0 | 27.8 | 27.6 | 23.2 | 24.0 |
| Jharkhand | 34.6 | 30.5 | 31.1 | 28.5 | 25.1 | 23.7 | 21.8 | 22.2 | 25.3 |
| Madhya Pradesh | 38.2 | 26.3 | 27.7 | 25.2 | 23.8 | 25.6 | 24.1 | 24.2 | 23.0 |
| Northeast | 36.3 | 34.3 | 34.6 | 31.5 | 25.7 | 26.7 | 27.5 | 24.4 | 24.5 |
| Odisha | 29.8 | 25.4 | 26.1 | 24.6 | 24.7 | 25.6 | 24.9 | 24.0 | 26.3 |
| Rajasthan | 27.3 | 23.6 | 24.0 | 24.7 | 24.8 | 24.3 | 21.8 | 21.3 | 24.2 |
| Uttar Pradesh | 36.3 | 33.1 | 30.9 | 33.4 | 29.8 | 28.6 | 28.2 | 27.5 | 30.9 |
| Andhra Pradesh | 28.5 | 22.6 | 22.1 | 19.5 | 24.5 | 25.8 | 26.1 | 27.7 | 28.0 |
| Delhi | 27.5 | 25.8 | 28.2 | 30.1 | 25.5 | 22.8 | 22.2 | 24.2 | 25.3 |
| Gujarat | 21.2 | 16.4 | 16.6 | 17.0 | 21.2 | 21.7 | 21.2 | 21.0 | 22.4 |
| Haryana | 33.7 | 30.9 | 31.5 | 33.0 | 31.4 | 29.1 | 27.1 | 24.6 | 26.5 |
| Karnataka | 26.8 | 20.6 | 20.8 | 19.8 | 24.4 | 24.6 | 24.8 | 25.4 | 26.3 |
| Kerala | 29.6 | 24.4 | 24.6 | 26.6 | 33.3 | 33.7 | 37.1 | 36.1 | 37.0 |
| Maharashtra | 25.0 | 17.5 | 17.9 | 17.6 | 22.9 | 24.5 | 25.8 | 24.8 | 25.0 |
| Punjab | 27.4 | 22.9 | 25.0 | 23.3 | 23.0 | 25.2 | 23.8 | 23.9 | 26.0 |
| Tamil Nadu | 34.4 | 26.0 | 26.0 | 23.6 | 27.8 | 30.7 | 31.1 | 31.8 | 33.2 |
| Telangana | 27.6 | 21.4 | 21.2 | 18.9 | 20.9 | 21.9 | 21.8 | 21.4 | 22.6 |
| West Bengal | 32.2 | 29.8 | 29.2 | 27.7 | 25.7 | 26.5 | 25.7 | 26.5 | 30.6 |

Table S4: Share of Watch formulations across years, state level

| states/years | 2011 | 2012 | 2013 | 2014 | 2015 | 2016 | 2017 | 2018 | 2019 |
| --- | --- | --- | --- | --- | --- | --- | --- | --- | --- |
| Bihar | 44.4 | 43.7 | 41.4 | 39.4 | 42.3 | 44.9 | 47.4 | 49.3 | 53.4 |
| Chhattisgarh | 45.9 | 49.6 | 44.7 | 44.8 | 46.3 | 49.5 | 50.3 | 53.6 | 56.7 |
| Jharkhand | 46.4 | 41.7 | 39.0 | 40.4 | 44.9 | 47.7 | 49.2 | 49.9 | 53.0 |
| Madhya Pradesh | 47.1 | 49.9 | 46.6 | 46.6 | 49.2 | 50.4 | 53.1 | 53.2 | 57.0 |
| Northeast | 50.4 | 47.8 | 46.3 | 45.7 | 50.5 | 52.8 | 51.9 | 54.7 | 57.8 |
| Odisha | 50.0 | 46.3 | 44.4 | 43.2 | 44.4 | 46.5 | 46.9 | 47.5 | 50.0 |
| Rajasthan | 55.4 | 51.5 | 49.2 | 46.8 | 47.7 | 50.6 | 53.3 | 54.3 | 56.9 |
| Uttar Pradesh | 48.9 | 46.7 | 46.9 | 43.9 | 46.3 | 49.6 | 51.1 | 51.2 | 50.3 |
| Andhra Pradesh | 52.9 | 51.2 | 50.1 | 50.8 | 50.7 | 53.4 | 53.7 | 52.4 | 53.2 |
| Delhi | 54.4 | 50.8 | 48.5 | 47.8 | 51.9 | 55.3 | 57.1 | 57.0 | 59.2 |
| Gujarat | 61.3 | 57.8 | 56.1 | 54.2 | 54.4 | 54.8 | 56.3 | 57.0 | 58.6 |
| Haryana | 51.4 | 48.5 | 47.1 | 44.6 | 47.9 | 52.7 | 55.0 | 58.4 | 58.3 |
| Karnataka | 56.9 | 53.0 | 51.6 | 50.9 | 51.5 | 53.6 | 53.0 | 53.7 | 55.6 |
| Kerala | 54.5 | 51.5 | 52.6 | 51.3 | 51.8 | 53.5 | 51.5 | 52.2 | 51.7 |
| Maharashtra | 59.0 | 55.9 | 54.3 | 53.5 | 55.1 | 55.1 | 55.3 | 56.5 | 59.2 |
| Punjab | 56.2 | 53.7 | 50.7 | 51.3 | 54.0 | 56.0 | 58.0 | 57.4 | 57.7 |
| Tamil Nadu | 52.6 | 51.9 | 50.8 | 52.5 | 52.3 | 52.1 | 53.4 | 53.4 | 52.6 |
| Telangana | 53.0 | 51.0 | 49.6 | 51.0 | 51.4 | 54.7 | 54.8 | 54.6 | 56.7 |
| West Bengal | 52.1 | 48.3 | 46.6 | 46.4 | 50.4 | 52.3 | 54.8 | 53.8 | 53.0 |

Table S5: Access to watch ratios across years, state level

| states/years | 2011 | 2012 | 2013 | 2014 | 2015 | 2016 | 2017 | 2018 | 2019 |
| --- | --- | --- | --- | --- | --- | --- | --- | --- | --- |
| Bihar | 0.86 | 0.75 | 0.78 | 0.83 | 0.64 | 0.61 | 0.55 | 0.49 | 0.46 |
| Chhattisgarh | 0.82 | 0.50 | 0.64 | 0.63 | 0.63 | 0.56 | 0.55 | 0.43 | 0.42 |
| Jharkhand | 0.75 | 0.73 | 0.80 | 0.71 | 0.56 | 0.50 | 0.44 | 0.45 | 0.48 |
| Madhya Pradesh | 0.81 | 0.53 | 0.59 | 0.54 | 0.48 | 0.51 | 0.45 | 0.46 | 0.40 |
| Northeast | 0.72 | 0.72 | 0.75 | 0.69 | 0.51 | 0.51 | 0.53 | 0.45 | 0.42 |
| Odisha | 0.60 | 0.55 | 0.59 | 0.57 | 0.56 | 0.55 | 0.53 | 0.50 | 0.53 |
| Rajasthan | 0.49 | 0.46 | 0.49 | 0.53 | 0.52 | 0.48 | 0.41 | 0.39 | 0.43 |
| Uttar Pradesh | 0.74 | 0.71 | 0.66 | 0.76 | 0.64 | 0.58 | 0.55 | 0.54 | 0.62 |
| Andhra Pradesh | 0.54 | 0.44 | 0.44 | 0.38 | 0.48 | 0.48 | 0.49 | 0.53 | 0.53 |
| Delhi | 0.51 | 0.51 | 0.58 | 0.63 | 0.49 | 0.41 | 0.39 | 0.42 | 0.43 |
| Gujarat | 0.35 | 0.28 | 0.30 | 0.31 | 0.39 | 0.39 | 0.38 | 0.37 | 0.38 |
| Haryana | 0.66 | 0.64 | 0.67 | 0.74 | 0.65 | 0.55 | 0.49 | 0.42 | 0.45 |
| Karnataka | 0.47 | 0.39 | 0.40 | 0.39 | 0.47 | 0.46 | 0.47 | 0.47 | 0.47 |
| Kerala | 0.54 | 0.47 | 0.47 | 0.52 | 0.64 | 0.63 | 0.72 | 0.69 | 0.72 |
| Maharashtra | 0.42 | 0.31 | 0.33 | 0.33 | 0.42 | 0.44 | 0.47 | 0.44 | 0.42 |
| Punjab | 0.49 | 0.43 | 0.49 | 0.45 | 0.43 | 0.45 | 0.41 | 0.42 | 0.45 |
| Tamil Nadu | 0.65 | 0.50 | 0.51 | 0.45 | 0.53 | 0.59 | 0.58 | 0.59 | 0.63 |
| Telangana | 0.52 | 0.42 | 0.43 | 0.37 | 0.41 | 0.40 | 0.40 | 0.39 | 0.40 |
| West Bengal | 0.62 | 0.62 | 0.63 | 0.60 | 0.51 | 0.51 | 0.47 | 0.49 | 0.58 |

Table S6: Share of Reserve formulations across years, state level

| states/years | 2011 | 2012 | 2013 | 2014 | 2015 | 2016 | 2017 | 2018 | 2019 |
| --- | --- | --- | --- | --- | --- | --- | --- | --- | --- |
| Bihar | 0.13 | 0.13 | 0.13 | 0.12 | 0.23 | 0.22 | 0.50 | 0.50 | 0.71 |
| Chhattisgarh | 0.17 | 0.25 | 0.25 | 0.25 | 0.35 | 0.39 | 0.57 | 0.60 | 0.76 |
| Jharkhand | 0.13 | 0.25 | 0.26 | 0.24 | 0.35 | 0.48 | 0.57 | 0.59 | 0.80 |
| Madhya Pradesh | 0.24 | 0.40 | 0.29 | 0.41 | 0.43 | 0.56 | 0.61 | 0.70 | 0.98 |
| Northeast | 0.25 | 0.44 | 0.44 | 0.53 | 0.53 | 0.66 | 0.96 | 1.03 | 1.31 |
| Odisha | 0.37 | 0.58 | 0.61 | 0.45 | 0.68 | 0.75 | 1.01 | 1.11 | 1.47 |
| Rajasthan | 0.37 | 0.48 | 0.50 | 0.57 | 0.62 | 0.75 | 0.95 | 1.13 | 1.28 |
| Uttar Pradesh | 0.19 | 0.28 | 0.28 | 0.28 | 0.43 | 0.51 | 0.59 | 0.67 | 0.89 |
| Andhra Pradesh | 0.22 | 0.32 | 0.46 | 0.46 | 0.46 | 0.77 | 0.92 | 0.99 | 1.20 |
| Delhi | 0.44 | 0.54 | 0.56 | 0.62 | 0.81 | 1.00 | 1.04 | 1.06 | 1.14 |
| Gujarat | 0.46 | 0.41 | 0.49 | 0.47 | 0.50 | 0.58 | 0.87 | 0.92 | 1.10 |
| Haryana | 0.24 | 0.44 | 0.56 | 0.59 | 0.53 | 0.59 | 0.85 | 0.98 | 1.28 |
| Karnataka | 0.17 | 0.31 | 0.37 | 0.36 | 0.42 | 0.57 | 0.78 | 0.86 | 0.96 |
| Kerala | 0.21 | 0.22 | 0.31 | 0.32 | 0.35 | 0.52 | 0.57 | 0.58 | 0.79 |
| Maharashtra | 0.24 | 0.32 | 0.34 | 0.32 | 0.54 | 0.58 | 0.75 | 0.82 | 0.94 |
| Punjab | 0.31 | 0.39 | 0.43 | 0.36 | 0.44 | 0.47 | 0.61 | 0.78 | 0.93 |
| Tamil Nadu | 0.18 | 0.29 | 0.32 | 0.33 | 0.54 | 0.77 | 0.85 | 1.02 | 1.19 |
| Telangana | 0.18 | 0.29 | 0.39 | 0.38 | 0.52 | 0.81 | 0.98 | 1.21 | 1.34 |
| West Bengal | 0.29 | 0.45 | 0.47 | 0.47 | 0.50 | 0.50 | 0.66 | 0.82 | 1.08 |

Table S7: Share of Discouraged FDCs across years, state level

| states/years | 2011 | 2012 | 2013 | 2014 | 2015 | 2016 | 2017 | 2018 | 2019 |
| --- | --- | --- | --- | --- | --- | --- | --- | --- | --- |
| Bihar | 16.9 | 20.0 | 22.2 | 23.9 | 29.8 | 27.2 | 25.8 | 26.0 | 21.1 |
| Chhattisgarh | 15.6 | 20.2 | 21.4 | 21.8 | 23.6 | 21.7 | 21.0 | 21.9 | 18.1 |
| Jharkhand | 18.1 | 22.9 | 25.6 | 26.0 | 29.0 | 27.4 | 27.8 | 26.6 | 20.4 |
| Madhya Pradesh | 13.7 | 17.7 | 19.7 | 21.7 | 26.0 | 22.8 | 21.6 | 21.5 | 18.6 |
| Northeast | 12.6 | 14.1 | 15.6 | 18.3 | 23.0 | 19.8 | 19.6 | 19.7 | 16.3 |
| Odisha | 18.8 | 23.0 | 24.2 | 26.4 | 29.6 | 26.3 | 26.3 | 26.5 | 21.4 |
| Rajasthan | 15.6 | 17.9 | 19.6 | 21.6 | 25.7 | 23.4 | 23.0 | 22.4 | 17.0 |
| Uttar Pradesh | 13.8 | 16.6 | 18.4 | 19.1 | 23.0 | 20.9 | 19.6 | 20.1 | 17.6 |
| Andhra Pradesh | 17.5 | 20.8 | 22.1 | 22.4 | 23.9 | 19.4 | 18.5 | 17.9 | 16.9 |
| Delhi | 16.0 | 17.4 | 17.9 | 16.5 | 20.5 | 19.8 | 18.7 | 16.9 | 13.7 |
| Gujarat | 15.0 | 18.6 | 20.2 | 21.2 | 22.5 | 21.5 | 20.2 | 19.8 | 17.0 |
| Haryana | 13.4 | 16.1 | 17.0 | 17.7 | 19.0 | 16.7 | 16.2 | 15.3 | 13.3 |
| Karnataka | 14.8 | 18.5 | 20.5 | 21.5 | 22.5 | 20.2 | 20.2 | 19.0 | 16.3 |
| Kerala | 14.3 | 16.2 | 15.8 | 15.2 | 13.6 | 11.4 | 10.0 | 10.3 | 9.8 |
| Maharashtra | 14.1 | 18.1 | 19.8 | 20.6 | 20.0 | 18.6 | 16.9 | 16.8 | 14.0 |
| Punjab | 14.4 | 17.3 | 18.7 | 19.8 | 21.0 | 17.2 | 16.4 | 16.8 | 14.7 |
| Tamil Nadu | 11.4 | 14.1 | 15.4 | 16.0 | 17.9 | 14.8 | 13.3 | 12.7 | 12.1 |
| Telangana | 18.3 | 22.0 | 23.3 | 23.3 | 26.0 | 21.3 | 21.1 | 21.6 | 18.3 |
| West Bengal | 14.3 | 17.9 | 20.3 | 21.1 | 22.4 | 19.9 | 18.3 | 18.4 | 14.9 |

Table S8: Share of Not classified formulations across years, state level

| states/years | 2011 | 2012 | 2013 | 2014 | 2015 | 2016 | 2017 | 2018 | 2019 |
| --- | --- | --- | --- | --- | --- | --- | --- | --- | --- |
| Bihar | 0.54 | 3.34 | 3.79 | 3.91 | 0.57 | 0.45 | 0.38 | 0.25 | 0.24 |
| Chhattisgarh | 0.84 | 4.89 | 4.98 | 5.16 | 0.70 | 0.62 | 0.57 | 0.60 | 0.48 |
| Jharkhand | 0.80 | 4.74 | 4.03 | 4.83 | 0.70 | 0.72 | 0.68 | 0.71 | 0.57 |
| Madhya Pradesh | 0.72 | 5.64 | 5.74 | 6.13 | 0.57 | 0.70 | 0.61 | 0.42 | 0.42 |
| Northeast | 0.51 | 3.39 | 3.16 | 4.06 | 0.21 | 0.11 | 0.12 | 0.13 | 0.15 |
| Odisha | 1.10 | 4.73 | 4.76 | 5.39 | 0.68 | 0.86 | 0.90 | 0.89 | 0.79 |
| Rajasthan | 1.35 | 6.54 | 6.69 | 6.26 | 1.15 | 0.97 | 0.95 | 0.88 | 0.64 |
| Uttar Pradesh | 0.77 | 3.33 | 3.53 | 3.38 | 0.52 | 0.43 | 0.49 | 0.48 | 0.30 |
| Andhra Pradesh | 0.97 | 5.05 | 5.32 | 6.92 | 0.55 | 0.67 | 0.74 | 0.99 | 0.65 |
| Delhi | 1.65 | 5.42 | 4.83 | 4.89 | 1.32 | 1.13 | 1.00 | 0.88 | 0.66 |
| Gujarat | 1.97 | 6.73 | 6.63 | 7.10 | 1.44 | 1.43 | 1.45 | 1.21 | 0.88 |
| Haryana | 1.21 | 4.05 | 3.86 | 4.14 | 1.22 | 0.95 | 0.94 | 0.73 | 0.51 |
| Karnataka | 1.45 | 7.51 | 6.75 | 7.39 | 1.20 | 1.14 | 1.20 | 1.01 | 0.81 |
| Kerala | 1.39 | 7.63 | 6.61 | 6.62 | 0.98 | 0.81 | 0.85 | 0.87 | 0.79 |
| Maharashtra | 1.70 | 8.21 | 7.66 | 8.09 | 1.44 | 1.23 | 1.24 | 1.07 | 0.86 |
| Punjab | 1.66 | 5.72 | 5.23 | 5.18 | 1.60 | 1.21 | 1.14 | 1.09 | 0.72 |
| Tamil Nadu | 1.38 | 7.70 | 7.54 | 7.55 | 1.52 | 1.68 | 1.34 | 1.15 | 0.92 |
| Telangana | 0.98 | 5.31 | 5.52 | 6.51 | 1.18 | 1.34 | 1.26 | 1.21 | 1.00 |
| West Bengal | 1.06 | 3.51 | 3.50 | 4.44 | 1.00 | 0.83 | 0.57 | 0.51 | 0.43 |

Table S9: Share of FDC formulations across years, state level

| states/years | 2011 | 2012 | 2013 | 2014 | 2015 | 2016 | 2017 | 2018 | 2019 |
| --- | --- | --- | --- | --- | --- | --- | --- | --- | --- |
| Bihar | 36.8 | 38.3 | 40.3 | 45.2 | 42.9 | 40.8 | 41.2 | 37.8 | 33.5 |
| Chhattisgarh | 44.9 | 38.5 | 43.2 | 44.0 | 44.9 | 41.8 | 41.8 | 37.1 | 33.3 |
| Jharkhand | 36.0 | 38.8 | 42.3 | 43.4 | 42.4 | 40.2 | 41.3 | 39.4 | 34.6 |
| Madhya Pradesh | 42.3 | 37.2 | 41.6 | 42.3 | 42.2 | 41.2 | 38.9 | 38.8 | 33.4 |
| Northeast | 39.2 | 40.4 | 42.7 | 44.8 | 43.3 | 41.6 | 42.7 | 39.6 | 36.1 |
| Odisha | 30.5 | 36.2 | 37.8 | 41.2 | 41.2 | 39.4 | 39.8 | 39.7 | 36.3 |
| Rajasthan | 34.2 | 37.3 | 40.0 | 42.3 | 43.5 | 40.9 | 41.6 | 39.9 | 34.8 |
| Uttar Pradesh | 34.1 | 34.4 | 34.4 | 37.4 | 38.0 | 35.2 | 34.4 | 34.1 | 34.1 |
| Andhra Pradesh | 32.4 | 32.6 | 34.6 | 36.9 | 38.7 | 34.9 | 34.5 | 35.1 | 34.4 |
| Delhi | 36.0 | 37.9 | 40.9 | 42.5 | 41.7 | 39.9 | 38.1 | 36.6 | 33.1 |
| Gujarat | 28.4 | 31.3 | 33.3 | 35.4 | 37.9 | 37.4 | 36.2 | 35.6 | 33.5 |
| Haryana | 34.3 | 35.0 | 37.0 | 38.3 | 37.0 | 34.0 | 35.6 | 32.3 | 29.9 |
| Karnataka | 32.6 | 34.2 | 35.8 | 37.2 | 40.8 | 40.3 | 40.5 | 39.7 | 37.8 |
| Kerala | 30.5 | 30.9 | 30.7 | 30.9 | 31.8 | 31.3 | 30.5 | 32.4 | 32.2 |
| Maharashtra | 31.4 | 33.0 | 34.6 | 36.6 | 37.6 | 36.6 | 35.0 | 35.1 | 33.2 |
| Punjab | 30.6 | 33.3 | 37.1 | 37.7 | 38.0 | 33.2 | 32.7 | 32.9 | 30.9 |
| Tamil Nadu | 31.5 | 29.1 | 31.0 | 31.5 | 36.3 | 34.8 | 34.4 | 34.4 | 32.4 |
| Telangana | 33.0 | 34.0 | 35.9 | 37.0 | 42.5 | 39.5 | 39.8 | 40.0 | 38.1 |
| West Bengal | 32.2 | 34.5 | 36.1 | 37.9 | 37.8 | 36.0 | 35.6 | 35.2 | 33.3 |

Table S10: Share of NLEM formulations across years, state level

| states/years | 2011 | 2012 | 2013 | 2014 | 2015 | 2016 | 2017 | 2018 | 2019 |
| --- | --- | --- | --- | --- | --- | --- | --- | --- | --- |
| Bihar | 41.8 | 40.0 | 40.1 | 39.0 | 39.0 | 40.8 | 44.5 | 44.6 | 48.7 |
| Chhattisgarh | 50.6 | 44.8 | 45.7 | 46.4 | 42.6 | 43.5 | 44.9 | 42.1 | 44.5 |
| Jharkhand | 48.7 | 43.6 | 41.8 | 40.8 | 40.5 | 42.1 | 42.7 | 44.1 | 49.0 |
| Madhya Pradesh | 49.9 | 45.4 | 44.1 | 43.1 | 40.2 | 42.9 | 43.8 | 44.6 | 45.8 |
| Northeast | 51.8 | 50.3 | 49.9 | 48.7 | 46.4 | 48.5 | 48.3 | 46.7 | 48.3 |
| Odisha | 43.2 | 40.0 | 40.2 | 38.6 | 44.1 | 45.7 | 46.1 | 45.8 | 49.4 |
| Rajasthan | 45.5 | 41.5 | 40.9 | 39.8 | 40.4 | 43.0 | 43.3 | 44.2 | 48.6 |
| Uttar Pradesh | 47.4 | 43.3 | 41.4 | 41.5 | 37.6 | 40.7 | 42.8 | 43.4 | 48.3 |
| Andhra Pradesh | 48.9 | 44.9 | 43.6 | 42.9 | 41.0 | 44.0 | 45.1 | 46.3 | 45.8 |
| Delhi | 44.7 | 42.8 | 44.9 | 46.8 | 47.1 | 47.3 | 48.7 | 51.4 | 53.4 |
| Gujarat | 43.6 | 39.5 | 37.9 | 37.5 | 43.4 | 44.4 | 45.8 | 46.6 | 49.5 |
| Haryana | 49.6 | 47.2 | 47.7 | 47.1 | 46.6 | 47.8 | 46.8 | 49.9 | 51.2 |
| Karnataka | 46.9 | 43.6 | 43.6 | 43.4 | 43.1 | 43.7 | 43.8 | 45.1 | 48.5 |
| Kerala | 51.3 | 48.0 | 48.7 | 51.7 | 55.1 | 54.5 | 56.8 | 56.4 | 59.9 |
| Maharashtra | 46.1 | 41.1 | 39.9 | 40.0 | 42.1 | 42.3 | 43.7 | 45.2 | 47.3 |
| Punjab | 50.6 | 47.4 | 46.7 | 45.7 | 43.0 | 47.3 | 47.7 | 47.8 | 51.3 |
| Tamil Nadu | 51.4 | 47.8 | 46.4 | 47.0 | 44.6 | 45.4 | 48.8 | 48.9 | 50.1 |
| Telangana | 48.1 | 43.8 | 42.7 | 42.5 | 38.6 | 39.8 | 40.9 | 40.6 | 43.2 |
| West Bengal | 48.4 | 45.5 | 43.6 | 44.0 | 44.5 | 45.1 | 48.9 | 48.3 | 52.0 |

Table S11: Share of CDSCO approved formulations across years, state level

| states/years | 2011 | 2012 | 2013 | 2014 | 2015 | 2016 | 2017 | 2018 | 2019 |
| --- | --- | --- | --- | --- | --- | --- | --- | --- | --- |
| Bihar | 46.8 | 46.8 | 47.0 | 45.4 | 43.3 | 46.1 | 47.7 | 48.2 | 51.5 |
| Chhattisgarh | 54.6 | 53.3 | 51.7 | 50.9 | 50.6 | 52.6 | 53.8 | 52.1 | 53.8 |
| Jharkhand | 50.2 | 50.8 | 47.1 | 45.9 | 45.4 | 48.4 | 48.6 | 50.4 | 53.9 |
| Madhya Pradesh | 55.4 | 55.2 | 52.9 | 51.1 | 48.6 | 51.3 | 52.3 | 54.1 | 56.2 |
| Northeast | 52.5 | 53.0 | 51.5 | 50.0 | 43.5 | 47.2 | 49.1 | 48.7 | 53.3 |
| Odisha | 45.3 | 45.0 | 44.6 | 44.4 | 42.8 | 46.9 | 48.1 | 48.5 | 50.9 |
| Rajasthan | 51.5 | 51.6 | 50.6 | 50.7 | 48.5 | 50.5 | 51.3 | 51.8 | 54.5 |
| Uttar Pradesh | 51.9 | 52.6 | 51.6 | 49.3 | 47.1 | 49.2 | 50.2 | 52.2 | 52.5 |
| Andhra Pradesh | 44.6 | 43.5 | 43.5 | 43.9 | 45.3 | 51.0 | 52.1 | 53.9 | 53.4 |
| Delhi | 49.4 | 50.9 | 51.4 | 52.8 | 50.1 | 50.1 | 49.6 | 49.7 | 50.8 |
| Gujarat | 47.6 | 47.2 | 45.8 | 46.5 | 47.4 | 47.8 | 47.4 | 48.3 | 49.9 |
| Haryana | 50.9 | 53.1 | 52.3 | 51.3 | 49.4 | 51.9 | 50.4 | 51.7 | 51.2 |
| Karnataka | 50.5 | 49.2 | 48.1 | 48.8 | 49.9 | 52.2 | 52.6 | 52.9 | 55.7 |
| Kerala | 50.9 | 51.5 | 51.7 | 52.6 | 51.9 | 51.0 | 54.1 | 54.2 | 54.2 |
| Maharashtra | 49.3 | 47.5 | 46.0 | 45.8 | 47.3 | 48.0 | 46.8 | 47.9 | 49.5 |
| Punjab | 49.5 | 49.5 | 48.8 | 47.2 | 46.5 | 49.6 | 49.3 | 50.1 | 51.8 |
| Tamil Nadu | 50.1 | 48.5 | 47.5 | 47.7 | 48.2 | 49.2 | 51.4 | 53.1 | 54.2 |
| Telangana | 43.8 | 42.9 | 43.1 | 43.5 | 43.4 | 49.2 | 48.7 | 49.3 | 50.8 |
| West Bengal | 43.2 | 45.4 | 44.8 | 45.5 | 43.5 | 46.8 | 47.6 | 49.3 | 53.1 |

| Table S12: Population weighted mean DID across years, state groups | | | | |  |  |  |  |  |
| --- | --- | --- | --- | --- | --- | --- | --- | --- | --- |
| state groups | 2011 | 2012 | 2013 | 2014 | 2015 | 2016 | 2017 | 2018 | 2019 |
| High Focus | 9.0 | 9.2 | 9.1 | 9.4 | 9.8 | 9.9 | 8.9 | 9.0 | 8.8 |
| Non high focus | 13 | 13.2 | 12.9 | 13 | 13.3 | 13.4 | 13 | 12.6 | 12.4 |
|  |  |  |  |  |  |  |  |  |  |
| Table S13: Population weighted mean share of Access formulations across years, state groups | | | | | | |  |  |  |
| state groups | 2011 | 2012 | 2013 | 2014 | 2015 | 2016 | 2017 | 2018 | 2019 |
| High Focus | 35.3 | 30.3 | 29.9 | 30.1 | 27.1 | 26.9 | 25.9 | 24.9 | 26.7 |
| Non high focus | 28.4 | 22.8 | 23 | 22.1 | 24.9 | 25.9 | 26.1 | 26.1 | 27.6 |
|  |  |  |  |  |  |  |  |  |  |
| Table S14: Population weighted mean share of Watch formulations across years, state groups | | | | | | |  |  |  |
| state groups | 2011 | 2012 | 2013 | 2014 | 2015 | 2016 | 2017 | 2018 | 2019 |
| High Focus | 48.6 | 47 | 45.4 | 43.7 | 46.2 | 48.9 | 50.6 | 51.5 | 53.3 |
| Non high focus | 55.4 | 52.6 | 51.2 | 50.9 | 52.4 | 53.8 | 54.7 | 55 | 55.9 |
|  |  |  |  |  |  |  |  |  |  |
| Table S15: Population weighted mean share of Reserve formulations across years, state groups | | | | | | |  |  |  |
| state groups | 2011 | 2012 | 2013 | 2014 | 2015 | 2016 | 2017 | 2018 | 2019 |
| High Focus | 0.22 | 0.32 | 0.31 | 0.33 | 0.43 | 0.51 | 0.67 | 0.74 | 0.97 |
| Non high focus | 0.26 | 0.35 | 0.41 | 0.4 | 0.5 | 0.62 | 0.79 | 0.89 | 1.1 |
|  |  |  |  |  |  |  |  |  |  |

| Table S16: Population weighted mean share of Discouraged FDCs across years, state groups | | | | | | |  |  |  |
| --- | --- | --- | --- | --- | --- | --- | --- | --- | --- |
| state groups | 2011 | 2012 | 2013 | 2014 | 2015 | 2016 | 2017 | 2018 | 2019 |
| High Focus | 15.1 | 18.2 | 20 | 21.4 | 25.6 | 23.2 | 22.3 | 22.4 | 18.6 |
| Non high focus | 14.5 | 17.9 | 19.4 | 20 | 21 | 18.5 | 17.3 | 17 | 14.7 |
|  |  |  |  |  |  |  |  |  |  |
| Table S17: Population weighted mean share of Not-classified formulations across years, state groups | | | | | | | | |  |
| state groups | 2011 | 2012 | 2013 | 2014 | 2015 | 2016 | 2017 | 2018 | 2019 |
| High Focus | 0.8 | 4.2 | 4.3 | 4.5 | 0.61 | 0.56 | 0.55 | 0.5 | 0.39 |
| Non high focus | 1.4 | 6.3 | 6 | 6.6 | 1.2 | 1.2 | 1.1 | 1 | 0.76 |
|  |  |  |  |  |  |  |  |  |  |
| Table S18: Population weighted mean share of FDC formulations across years, state groups | | | | | | |  |  |  |
| state groups | 2011 | 2012 | 2013 | 2014 | 2015 | 2016 | 2017 | 2018 | 2019 |
| High Focus | 36.3 | 36.7 | 38.6 | 41.3 | 41.1 | 38.8 | 38.6 | 37.1 | 34.3 |
| Non high focus | 31.7 | 32.8 | 34.7 | 36.1 | 38 | 36.3 | 35.7 | 35.5 | 33.6 |
|  |  |  |  |  |  |  |  |  |  |
| Table S19: Population weighted mean share of NLEM formulations across years, state groups | | | | | | |  |  |  |
| state groups | 2011 | 2012 | 2013 | 2014 | 2015 | 2016 | 2017 | 2018 | 2019 |
| High Focus | 46.8 | 43.2 | 42.2 | 41.5 | 39.9 | 42.4 | 44 | 44.2 | 48 |
| Non high focus | 47.9 | 44.2 | 43.3 | 43.4 | 43.8 | 44.8 | 46.5 | 47.2 | 49.6 |
|  |  |  |  |  |  |  |  |  |  |
| Table S20: Population weighted mean share of CDSCO approved formulations across years, state groups | | | | | | | |  |  |
| state groups | 2011 | 2012 | 2013 | 2014 | 2015 | 2016 | 2017 | 2018 | 2019 |
| High Focus | 51 | 51.3 | 50.1 | 48.6 | 46.3 | 48.8 | 50 | 51.1 | 53.1 |
| Non high focus | 47.9 | 47.5 | 46.7 | 47 | 47.1 | 49.2 | 49.5 | 50.6 | 52.2 |

Figure S2: Time trends in FDCs, NLEM formulations, and CDSCO-approved products across HF and nHF states

*Note: HF— high focus, nHF— non high focus, FDC— fixed dose combinations, NLEM— national list of essential medicines, CDSCO— Central Drugs Standard Control Organization*
